# Access to Reproductive Health Services Among People With Disabilities

**DOI:** 10.1001/jamanetworkopen.2023.44877

**Published:** 2023-11-29

**Authors:** M. Antonia Biggs, Rosalyn Schroeder, M. Tara Casebolt, Bianca I. Laureano, Robin L. Wilson-Beattie, Lauren J. Ralph, Shelly Kaller, Aliza Adler, Margaret W. Gichane

**Affiliations:** 1 Advancing New Standards in Reproductive Health (ANSIRH), Bixby Center for Global Reproductive Health, Department of Obstetrics, Gynecology, and Reproductive Sciences, University of California San Francisco, San Francisco; 2 Morrissey College of Arts and Sciences, Boston College, Chestnut Hill, Massachusetts; 3 Independent scholars; 4 Innovating Education in Reproductive Health, Bixby Center for Global Reproductive Health, Department of Obstetrics, Gynecology, and Reproductive Sciences, University of California San Francisco, San Francisco

## Abstract

**Importance:**

People with disabilities face inequitable access to reproductive health (RH) services, yet the national prevalence of barriers to access experienced across disability types and statuses is unknown.

**Objective:**

To assess the national prevalence of barriers to RH access experienced by people with disabilities.

**Design, Setting, and Participants:**

This cross-sectional study analyzed results of an online probability-based national survey of RH experiences that was conducted from December 2021 to January 2022. The national sample consisted of English- and Spanish-speaking panel members of a market research firm. Panelists were invited to participate in a survey on their RH experiences and opinions. These participants were aged 15 to 49 years and assigned female at birth (AFAB). Weighted proportions were estimated, and bivariable and multivariable regression analyses were performed to assess associations between disability status and barriers to accessing RH services.

**Exposure:**

Using 5 of the 6 Washington Group Short Set on Functioning items, 8 dichotomous disability indicators were created: (1) vision, (2) hearing, (3) mobility, (4) activities of daily living, (5) communication, (6) overall disability status (a lot or more difficulty functioning in ≥1 domain), (7) some difficulty functioning (below the disability threshold; some or more difficulty functioning in ≥1 domain), and (8) multiple disabilities (a lot or more difficulty functioning in ≥2 domains).

**Main Outcomes and Measures:**

Number and types of barriers (logistical, access, cost, privacy, and interpersonal relationship) to accessing RH services in the past 3 years.

**Results:**

After exclusion, the final sample included 6956 people AFAB, with a mean (SD) age of 36.0 (8.3) years. Of these participants, 8.5% (95% CI, 7.6%-9.5%) met the disability threshold. Participants with disabilities compared with those without disabilities were disproportionately more likely to be non-Hispanic Black (18.8% [95% CI, 14.4%-24.1%] vs 13.2% [95% CI, 11.9%-14.5%]) or Hispanic or Latinx (completed survey in English: 18.1% [95% CI, 14.0%-23.0%] vs 14.6% [95% CI, 13.3%-16.0%]; completed survey in Spanish: 8.9% [95% CI, 6.2%-12.8%] vs 6.2% [95% CI, 5.4%-7.1%]) individuals, to identify as LGBTQAI (lesbian, gay, bisexual, transgender, queer [or questioning], asexual [or allied], intersex; 16.4% [95% CI, 12.3%-21.6%] vs 11.8% [95% CI, 10.6%-13.1%]), to live below the federal poverty level (27.3% [95% CI, 22.3%-32.8%] vs 10.7% [95% CI, 9.7%-11.9%]), and to ever experienced medical mistreatment (49.6% [95% CI, 43.7%-55.5%] vs 36.5% [95% CI, 34.8%-38.2%]). Among those who had ever tried to access RH services (n = 6027), people with disabilities vs without disabilities were more likely to experience barriers (69.0% [95% CI, 62.9%-74.5%] vs 43.0% [95% CI, 41.2%-44.9%]), which were most often logistical (50.7%; 95% CI, 44.2%-57.2%) and access (49.9%; 95% CI, 43.4%-56.4%) barriers. The disability domains with the highest proportion of people who experienced 3 or more barriers in the past 3 years included activities of daily living (75.3%; 95% CI, 61.1%-85.6%), communication (65.1%; 95% CI, 49.5%-78.1%), and multiple (59.9%; 95% CI, 45.6%-72.7%) disabilities.

**Conclusions and Relevance:**

This cross-sectional study found large disparities in access to RH services among people AFAB with disabilities. Findings indicated a need to alleviate barriers to RH care, including improving the transportation infrastructure and reinforcing patient-centered approaches that engender inclusivity in health care settings.

## Key Points

**Question:**

What is the prevalence of barriers to reproductive health (RH) access in the US among people with disabilities by disability type and status?

**Findings:**

In this cross-sectional study of 6956 people assigned female at birth, people with disabilities across disability domains (hearing, vision, mobility, activities of daily living, and communication) and status experienced considerably more barriers in trying to access RH care in the past 3 years compared with people without disabilities.

**Meaning:**

Findings of this study suggest a need to improve the transportation infrastructure and reinforce patient-centered approaches to engender inclusivity in health care settings.

## Teaser

This cross-sectional study examines the logistical, access, cost, privacy, and interpersonal barriers to reproductive health care in females with varying disabilities and levels of functioning.

## Introduction

The Americans with Disability Act (ADA) defines disability as a “physical or mental impairment that substantially limits 1 or more major life activity.”^1^ Levels of difficulty functioning can range in severity and span across multiple domains, each requiring unique accommodations to ensure adequate access and provision of high-quality health care, including sexual and reproductive health (RH) services. People with disabilities are disproportionately more likely than those without disabilities to have poor health, more health care needs, and gynecological cancers yet are less likely to receive gynecological cancer screenings and timely and consistent prenatal care, although they are similarly likely to get pregnant.^2,3,4,5,6,7,8,9,10,11^ Although the ADA requires equal access to health care for people with disabilities, inequities persist.^12^ Understanding the barriers that prevent people with disabilities from accessing RH services is critical to identifying inequities and informing patient-centered approaches to services.

Ableism, the practice of giving privilege to able-bodied people, adversely affects people’s access to and experiences with RH services as well as their reproductive well-being.^13^ Examples of ableism in RH settings include lack of health care practitioner training on caring for people with disabilities, disability stigma, inaccessible health care facilities, nonexistent adaptive equipment and skilled language interpreters, and overall substandard quality of care.^13,14,15,16,17,18,19,20,21,22^ Patients have described receiving poor-quality contraceptive care due to practitioners’ biased assumptions that people with disabilities are not sexually active and a lack of knowledge and insensitivity regarding management of contraceptive methods and their adverse effects alongside their disability.^20,21,22^ Experiences of discrimination, exclusion, and stigma, paired with a health care infrastructure that does not adequately address the needs of people with disabilities, have likely contributed to substantial disparities in RH outcomes. People assigned female at birth (AFAB) with disabilities experience higher rates of intimate partner violence, reproductive coercion, unintended pregnancy, cervical and breast cancers, and poor birth outcomes than their counterparts.^2,20,23,24,25,26^

Small qualitative studies have described the challenges that people with disabilities experience when trying to access RH services. These studies have found that people with disabilities often lack insurance for preferred contraceptive methods, lack privacy when discussing sensitive topics (eg, contraception) due to the presence of caregivers, and lack resources and information about RH screenings, which affect their adherence to screening protocols.^20,21,22^ However, the extent to which people with disabilities experience insurance, privacy-related, and other barriers is unknown.

Limitations of studies that have examined the barriers experienced by people with disabilities include small sample sizes, no assessment of the barrier types experienced, a focus on a sample of pregnant people rather than preventive RH care, and minimal recent evidence. Given the increase in barriers to RH access since the COVID-19 pandemic, particularly among historically structurally marginalized populations,^27^ an examination of the barriers experienced by people with disabilities is warranted. The current study aimed to assess the national prevalence of barriers to RH access experienced by people with disabilities, thereby filling an important research gap. We also examined the same barriers documented among the general public between 2017 and 2022.^27^ Using these previous data allowed us to compare the prevalence of access barriers among people with disabilities vs the general public. For this study, the 2 primary research questions were (1) what is the prevalence of barriers in trying to access RH services among people with disabilities? and (2) do the barriers vary by disability status and disability type? By surveying people from December 2021 to January 2022 regarding barriers to RH access in the past 3 years, we captured their experiences during the COVID-19 pandemic. We hypothesized that people with disabilities, particularly those with multiple disabilities, experience more barriers to care.

## Methods

### Study Design

This cross-sectional study analyzed results of an online, probability-based national survey, which was designed to assess the prevalence of attempts to self-manage an abortion, attitudes toward self-managed abortion, and interest in alternative models of medication abortion provision. The University of California San Francisco Institutional Review Board approved this study. Participants provided electronic informed consent before taking the survey. We followed the Strengthening the Reporting of Observational Studies in Epidemiology (STROBE) reporting guideline.

From December 2021 to January 2022, we fielded a large national survey to English- and Spanish-speaking, reproductive-aged (ages 15-49 years) people assigned female or male at birth; this analysis included only those AFAB. A market research firm administered the survey to their panel members (KnowledgePanel; Ipsos), using probability-based sampling techniques so that panelist recruitment was representative of the noninstitutionalized, English- and Spanish-speaking population living in the US when survey weights were applied.^28^ Panelists were invited to participate in a survey on their RH experiences and opinions. The survey collected data on barriers to accessing RH services, health care experiences, abortion attitudes, and sociodemographic characteristics. Automatic reminders were sent to nonresponders 3 and 8 days after the initial survey invitation. Participants were reimbursed through a points program, whereby they received cash-equivalent checks in amounts reflecting their level of panel participation, which commonly amounted to $4 to $6 per month.

### Outcome Variables

The primary outcomes were the number and types of barriers when trying to access RH services in the past 3 years, using previously published items.^27,29^ Reproductive health services were defined as “a Pap smear, which is a test to check for cervical cancer, or family planning, like birth control methods.” Those who had ever tried to access RH services were asked to select the barriers they had experienced in the past 3 years from a predefined list of 10 barriers: finding transportation to an office or clinic, getting time off work or school to go to the appointment, finding childcare so I could go to the office or clinic, finding a place that offers RH services, finding a place where I felt comfortable, finding services with people who speak my language, paying for services, finding a place that accepts my insurance, getting services without telling people you did not want to tell, and going to the clinic because my partner or family member did not want me to go. We created a 4-part categorical variable for the number of barriers experienced (none, 1, 2, and ≥3) and grouped barriers into 5 conceptual themes (logistical, access, cost, privacy, and interpersonal relationship), consistent with a previous study.^27^

### Independent Variables

We created 8 disability indicators as the primary exposures, using 5 of the 6 Washington Group Short Set (WG-SS) on Functioning items^30^: vision: do you have difficulty seeing?; hearing: do you have difficulty hearing, even when using hearing aids?; mobility: do you have difficulty walking or climbing stairs?; activities of daily living (ADLs): do you have difficulty with self-care, such as dressing or bathing?; and communication: using your usual language, do you have difficulty communicating, such as understanding or being understood? We excluded the cognition item, which asked how much difficulty do you have remembering or concentrating?, due to survey length constraints, overlap with the attention-deficit/hyperactivity disorder measure, and changes to the analytic plan. We changed the analytic plan to focus on WG-SS items, which were based on self-reported activity limitations, instead of including formal mental health diagnoses from a health care practitioner. For each item, the Likert-type answer options included no difficulty, some difficulty, a lot of difficulty, and cannot do at all. Following WG-SS criteria, participants who reported a lot of difficulty or cannot do at all in 1 or more domain were considered to have a disability (overall disability status) or a domain-specific disability (vision, hearing, mobility, ADLs, and communication).^31^ We also lowered the cutoff to measure some difficulty functioning (defined as some or more difficulty functioning in ≥1 domain) and included a measure of multiple disabilities (defined as a lot or more difficulty functioning in ≥2 domains), following WG-SS criteria.

### Statistical Analysis

For all analyses, we used sampling weights to produce estimates that were representative of the noninstitutionalized US population AFAB, based on US Census data. Design weights accounted for any differential nonresponse. We estimated weighted proportions and conducted χ^2^ analyses by overall disability status and participant characteristics (Table 1), by attempts to access RH services and domain-specific disability (Table 2), and by number (Figure) and types of barriers when trying to access RH services and disability indicators (Table 3). We conducted unadjusted and adjusted log-binomial regression analyses to assess associations between disability indicators and types of barriers experienced when trying to access RH services (Table 4). Covariates were selected a priori. We adjusted for age group (15-17, 18 and 19, 20-24, 25-29, 30-39, and 40-49 years), self-reported race and ethnicity and survey language (Hispanic or Latinx, who completed the survey in English; Hispanic or Latinx, who completed the survey in Spanish; non-Hispanic Asian, Native Hawaiian, or Pacific Islander [hereafter Asian]; non-Hispanic Black [hereafter Black]; non-Hispanic White [hereafter White]; and >1 non-Hispanic race or Other [American Indian or Alaska Native, Middle Eastern, North African, and people who selected Other], all of whom completed the survey in English), US nativity status, educational level, marital status (married; divorced, widowed, or separated; or never married), federal poverty level (<100%, 100%-199%, or ≥200%), metropolitan statistical area, geographic region of residence, LGBTQAI (lesbian, gay, bisexual, transgender, queer [or questioning], asexual [or allied], intersex and/or gender nonconforming) identity, medical mistreatment (when seeking health care, physicians, nurses, or other health care professionals made you feel your symptoms were not real, not severe, or not important, or made you feel ridiculed or humiliated), quality of medical care in the past year, and difficulty trusting health care physicians, nurses, or other health care professionals (ranging from not difficult to extremely difficult). Race and ethnicity were included in the analysis to ascertain whether structurally marginalized populations were disproportionately more likely to live with a disability and experience barriers when trying to access RH services.

**Table 1.** Demographic Characteristics and Health Care Experiences by Disability Status Among Survey Participants Assigned Female at Birth

Characteristic	Overall disability status				*P* value^a^	Total	
	Without disability		With disability				
	Weighted proportion (95% CI)	Raw No.	Weighted proportion (95% CI)	Raw No.		Weighted proportion (95% CI)	Raw No.
**Demographic characteristics**							
Total No.	100	6337	100	619	NA	100	6956
Race and ethnicity and survey language^b^							
Hispanic or Latinx; completed survey in English	14.6 (13.3-16.0)	834	18.1 (14.0-23.0)	110	.01	14.9 (13.6-16.2)	944
Hispanic or Latinx; completed survey in Spanish	6.2 (5.4-7.1)	389	8.9 (6.2-12.8)	57		6.5 (5.7-7.3)	446
Non-Hispanic Asian, Native Hawaiian, or Pacific Islander	5.9 (5.0-7.0)	226	3.4 (1.8-6.1)	19		5.7 (4.9-6.7)	245
Non-Hispanic Black	13.2 (11.9-14.5)	518	18.8 (14.4-24.1)	74		13.6 (12.4-15.0)	592
Non-Hispanic White	55.4 (53.6-57.2)	4147	46.9 (41.1-52.9)	340		54.7 (52.9-56.4)	4487
>1 Non-Hispanic race or Other^c^	4.7 (4.0-5.6)	223	3.9 (2.1-7.0)	19		4.6 (3.9-5.5)	242
Non–US nativity	14.3 (13.1-15.6)	819	19.1 (15.0-24.0)	109	.03	14.7 (13.5-16.0)	928
Age group, mean (SD), y	36.0 (8.3)		36.8 (8.4)		.29	36.0 (8.3)	
15-17	9.4 (7.9-11.0)	165	5.6 (2.8-11.0)	10	.87	9.0 (7.7-10.6)	175
18 and 19	2.4 (1.8-3.2)	50	3.7 (1.8-7.5)	8		2.5 (2.0-3.3)	58
20-24	12.6 (11.3-14.0)	337	16.0 (11.5-21.7)	35		12.9 (11.6-14.3)	372
25-29	15.6 (14.4-16.8)	943	16.1 (12.3-20.8)	78		15.6 (14.5-16.8)	1021
30-39	31.4 (29.8-32.9)	2433	30.6 (25.6-36.0)	218		31.3 (29.8-32.8)	2651
40-49	28.7 (27.2-30.2)	2409	28.0 (23.5-33.0)	270		28.6 (27.2-30.1)	2679
Educational level							
<High school	7.2 (6.2-8.3)	217	16.1 (12.0-21.3)	50	<.001	7.9 (6.9-9.1)	267
High school diploma	21.0 (19.4-22.6)	776	26.0 (20.9-31.8)	127		21.4 (19.9-23.0)	903
Some college	29.4 (27.8-31.1)	1722	32.5 (27.3-38.2)	214		29.7 (28.1-31.3)	1936
≥Bachelor’s degree	42.4 (40.7-44.1)	3622	25.3 (20.9-30.4)	228		40.9 (39.3-42.6)	3850
Marital status							
Married	55.5 (53.8-57.3)	3393	40.2 (34.6-46.0)	254	<.001	54.2 (52.5-55.9)	3647
Widow, divorced, or separated	7.0 (6.2-8.0)	629	11.5 (8.3-15.7)	102		7.4 (6.6-8.3)	731
Never married	37.4 (35.7-39.2)	2315	48.3 (42.5-54.3)	263		38.3 (36.7-40.0)	2578
FPL, %							
<100	10.7 (9.7-11.9)	894	27.3 (22.3-32.8)	217	<.001	12.1 (11.1-13.3)	1111
100-199	13.9 (12.7-15.3)	983	15.5 (11.9-19.9)	122		14.1 (12.9-15.3)	1105
≥200	75.3 (73.7-76.9)	4460	57.3 (51.4-63.0)	280		73.8 (72.2-75.3)	4740
MSA							
Nonmetropolitan area	11.6 (10.5-12.8)	816	12.0 (8.5-16.6)	84	.86	11.7 (10.6-12.8)	900
Metropolitan area	88.4 (87.2-89.5)	5521	88.0 (83.4-91.5)	535		88.3 (87.2-89.4)	6056
Geographic region of residence							
Northeast	16.8 (15.5-18.1)	983	15.8 (12.0-20.6)	96	.16	17.0 (15.4-18.0)	1079
Midwest	20.7 (19.3-22.1)	1640	15.8 (11.7-21.0)	118		20.0 (18.9-21.6)	1758
South	38.1 (36.3-39.9)	2225	43.9 (38.2-49.8)	261		39.0 (36.9-40.3)	2486
West	24.5 (23.0-26.1)	1489	24.4 (19.7-29.8)	144		25.0 (23.0-26.0)	1633
LGBTQAI identity	11.8 (10.6-13.1)	708	16.4 (12.3-21.6)	91	.03	12.1 (11.0-13.4)	799
Gay, lesbian, or queer	11.7 (10.5-13.0)	687	17.6 (13.2-23.1)	91	.01	12.2 (11.0-13.4)	778
Transgender or gender nonconforming	2.0 (1.5-2.6)	104	2.3 (1.0-5.1)	14	.71	2.0 (1.5-2.6)	118
**History of pregnancy and abortion and considering SMA**							
Pregnancy history							
Never been pregnant	46.0 (44.2-47.8)	2472	50.6 (44.6-56.4)	239	.04	46.4 (44.6-48.1)	2711
Been pregnant, never considered SMA	51.4 (49.6-53.2)	3691	45.0 (39.3-50.8)	346		51.8 (49.1-52.6)	4037
Been pregnant, considered SMA	2.6 (2.1-3.3)	164	4.5 (2.7-7.4)	29		2.8 (2.3-3.4)	193
History of abortion	10.9 (9.9-12.0)	740	8.9 (6.2-12.6)	66	.26	10.8 (9.8-11.8)	806
**Health care experiences**							
Ever tried to access RH services	78.4 (76.6-80.1)	5522	73.2 (67.1-78.4)	505	.07	77.9 (76.2-79.6)	6027
Difficulty trusting health care practitioners							
Extremely difficult	2.7 (2.2-3.3)	181	10.7 (7.4-15.3)	58	<.001	3.4 (2.8-4.1)	239
Very difficult	4.2 (3.5-5.0)	263	10.5 (7.5-14.4)	70		4.7 (4.0-5.5)	333
Somewhat difficult	18.0 (16.7-19.4)	1216	25.6 (20.8-31.1)	162		18.6 (17.3-20.0)	1378
Slightly difficult	22.5 (20.1-23.0)	1444	19.8 (15.4-25.1)	125		21.3 (20.0-22.8)	1569
Not difficult	53.6 (51.8-55.4)	3223	33.3 (28.1-39.1)	203		51.9 (50.2-53.6)	3426
Any medical mistreatment: when seeking health care, practitioners made you feel	36.5 (34.8-38.2)	2604	49.6 (43.7-55.5)	337	<.001	37.6 (36.0-39.2)	2941
Ridiculed or humiliated	19.4 (18.1-20.8)	1440	34.5 (29.0-40.3)	236	<.001	20.7 (19.4-22.0)	1676
Symptoms not real, not severe, or not important	32.6 (31.1-34.3)	2345	45.7 (39.9-51.6)	307	<.001	33.8 (32.2-35.4	2652
Quality of medical care from regular physician in past year							
Excellent	22.7 (21.2-24.2)	1399	21.8 (17.4-27.0)	142	<.001	22.6 (21.2-24.1)	1541
Very good	32.6 (30.9-34.3)	2059	22.3 (18.0-27.4)	151		31.7 (30.2-33.3)	2210
Good	22.5 (21.0-24.1)	1417	26.2 (21.1-32.1)	141		22.8 (21.4-24.4)	1558
Fair or poor	7.0 (6.2-8.0)	476	16.1 (12.2-21.0)	97		7.8 (6.9-8.7)	573
Have not seen my regular physician in past year	9.5 (8.5-10.6)	616	6.5 (4.2-10.0)	43		9.2 (8.3-10.3)	659
Do not have a regular physician	5.7 (4.9-6.6)	358	7.0 (4.6-10.4)	43		5.8 (5.1-6.7)	401

Abbreviations: FPL, federal poverty level; LGBTQAI, lesbian, gay, bisexual, transgender, queer (or questioning), asexual (or allied), intersex; MSA, metropolitan statistical area; NA, not applicable; RH, reproductive health; SMA, self-managed abortion.

^a^ *P* values were calculated using χ^2^ analysis.

^b^ Race and ethnicity were self-reported in the survey. Survey was completed in English unless otherwise specified.

^c^ Other race included American Indian or Alaska Native, Middle Eastern, North African, and people who selected Other.

**Table 2.** Association Between Domain-Specific Disability Level and Attempt to Access Reproductive Health (RH) Services in Past 3 Years Among Survey Participants Assigned Female at Birth

Domain-specific disability level	Participants who ever tried to access RH services in past 3 y, weighted proportion (95% CI)		Total (n = 6956)	
	No (n = 692)	Yes (n = 6027)	Weighted proportion (95% CI)	Raw No.
Vision				
No difficulty	73.3 (69.2-77.1)	74.6 (73.0-76.1)	74.3 (72.8-75.8)	5090
Some difficulty	20.0 (16.7-23.8)	21.0 (19.6-22.5)	20.8 (19.5-22.2)	1515
A lot of difficulty	4.8 (3.3-7.0)	3.3 (2.7-4.0)	3.6 (3.0-4.4)	257
Cannot do at all	1.8 (1.0-3.3)	1.0 (0.7-1.5)	1.2 (0.9-1.7)	82
Hearing				
No difficulty	92.4 (89.8-94.3)	91.1 (90.0-92.1)	91.4 (90.4-92.3)	6247
Some difficulty	5.6 (3.9-8.0)	7.4 (6.5-8.4)	7.0 (6.2-7.9)	565
A lot of difficulty	1.4 (0.7-2.7)	1.0 (0.6-1.4)	1.0 (0.7-1.5)	69
Cannot do at all	0.6 (0.3-1.4)	0.6 (0.3-1.0)	0.6 (0.4-0.9)	44
Mobility: walking or climbing stairs				
No difficulty	88.4 (85.4-90.9)	85.3 (83.9-86.5)	86.0 (84.8-87.1)	5840
Some difficulty	8.7 (6.5-11.5)	12.1 (11.0-13.3)	11.4 (10.3-12.5)	869
A lot of difficulty	2.2 (1.3-3.7)	2.1 (1.7-2.7)	2.2 (1.7-2.7)	194
Cannot do at all	0.7 (0.3-1.6)	0.5 (0.3-0.8)	0.5 (0.3-0.8)	43
ADLs: bathing or dressing				
No difficulty	93.6 (91.2-95.4)	94.3 (93.4-95.1)	94.2 (93.3-94.9)	6509
Some difficulty	3.9 (2.5-6.0)	4.5 (3.8-5.4)	4.4 (3.7-5.2)	340
A lot of difficulty	2.0 (1.2-3.5)	0.9 (0.6-1.4)	1.2 (0.9-1.6)	77
Cannot do at all	0.4 (0.2-1.2)	0.2 (0.1-0.5)	0.3 (0.2-0.5)	22
Communication: understanding or being understood				
No difficulty	88.3 (85.1-90.9)	93.0 (92.1-93.9)	92.0 (91.0-92.9)	6433
Some difficulty	9.1 (6.7-12.1)	5.4 (4.6-6.3)	6.2 (5.4-7.1)	404
A lot of difficulty	1.2 (0.6-2.5)	1.1 (0.8-1.6)	1.1 (0.8-1.6)	72
Cannot do at all	1.3 (0.7-2.5)	0.4 (0.3-0.8)	0.6 (0.4-1.0)	37
Some difficulty functioning: some or more difficulty functioning in ≥1 domain	38.4 (34.1-42.9)	37.4 (35.7-39.1)	37.6 (35.9-39.3)	2765
Overall disability status: a lot or more difficulty functioning in ≥1 domain	10.3 (8.0-13.2)	8.0 (7.0-9.0)	8.5 (7.6-9.5)	619
Multiple disabilities: a lot or more difficulty functioning in ≥2 domains	3.3 (2.2-5.0)	1.8 (1.4-2.4)	2.1 (1.7-2.7)	156

Abbreviation: ADLs, activities of daily living.

**Figure.**
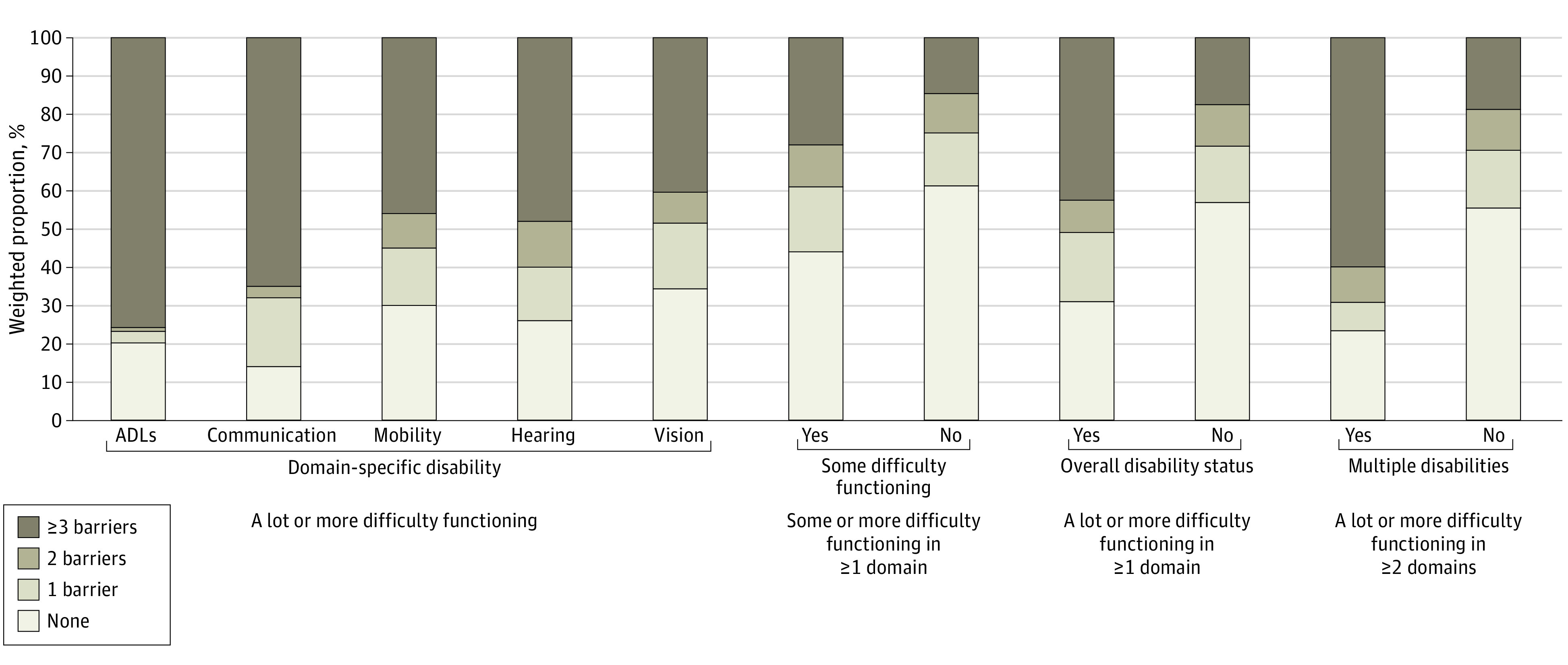
Number of Barriers to Reproductive Health Services in Past 3 Years by Disability Indicators Among Survey Participants Assigned Female at Birth (n = 6027) All differences by level of difficulty functioning and number of barriers had *P* < .001 according to weighted χ^2^ analyses. ADLs indicates activities of daily living.

**Table 3.** Barriers to Reproductive Health (RH) Services in the Past 3 Years by Disability Indicators Among Survey Participants Assigned Female at Birth Who Ever Tried to Access RH (N = 6027)^a^

Types of barriers to RH services	Disability indicator, weighted proportion (95% CI)						Total (n = 6027)	
	Some difficulty functioning^b^		Overall disability status^c^		Multiple disabilities^d^			
	No (n = 3648)	Yes (n = 2379)	Without disability (n = 5522)	With disability (n = 505)	No (n = 5910)	Yes (n = 117)	Weighted proportion (95% CI)	Raw No.
Had difficulty in past 3 y								
Logistical	26.7 (24.7-28.8)	39.1 (36.2-41.9)	29.7 (28.0-31.4)	50.7 (44.2-57.2)	30.7 (29.0-32.4)	66.2 (53.1-77.2)	31.3 (29.7-33.0)	1903
Finding transportation to an office or clinic	7.4 (6.1-8.8)	16.5 (14.4-18.9)	9.0 (7.9-10.2)	31.5 (25.4-38.2)	10.0 (8.9-11.3)	50.4 (36.5-64.1)	10.8 (9.6-12.0)	582
Getting time off work or school to go to the appointment	21.2 (19.3-23.1)	30.5 (27.9-33.4)	23.4 (21.8-25.0)	39.4 (33.0-46.2)	24.1 (22.6-25.7)	55.0 (41.2-68.0)	24.7 (23.1-26.3)	1442
Finding child care so I could go to the office or clinic	11.3 (10.0-12.9)	16.4 (14.4-18.6)	12.5 (11.3-13.8)	21.9 (17.0-27.8)	12.8 (11.7-14.1)	34.9 (22.7-49.4)	13.2 (12.1-14.5)	808
Access	22.6 (20.7-24.6)	36.3 (33.5-39.1)	25.8 (24.2-27.5)	49.9 (43.4-56.4)	27.1 (25.5-28.7)	63.4 (49.1-75.7)	27.7 (26.1-29.4)	1703
Finding a place that offers RH services	7.6 (6.5-8.9)	15.2 (13.2-17.5)	9.2 (8.2-10.3)	25.2 (19.9-31.4)	9.9 (8.9-11.1)	40.2 (27.2-54.8)	10.5 (9.4-11.6)	609
Finding a place where I felt comfortable	20.3 (18.5-22.2)	32.3 (29.7-35.1)	23.0 (21.5-24.6)	45.4 (39.0-52.1)	24.2 (22.7-25.8)	57.2 (43.0-70.4)	24.8 (23.3-26.4)	1544
Finding services with people who speak my language	5.5 (4.5-6.8)	12.3 (10.4-14.4)	6.8 (5.9-7.9)	22.1 (16.9-28.2)	7.5 (6.5-8.5)	40.1 (27.0-54.7)	8.0 (7.1-9.1)	412
Cost	19.0 (17.3-20.9)	34.0 (31.3-36.8)	22.8 (21.3-24.4)	45.6 (39.1-52.2)	24.1 (22.6-25.6)	53.9 (39.8-67.4)	24.6 (23.1-26.2)	1551
Paying for services	12.4 (11.0-14.0)	22.3 (19.9-24.8)	16.3 (15.0-17.7)	29.3 (23.7-35.6)	17.0 (15.6-18.3)	39.0 (26.3-53.4)	17.3 (16.0-18.7)	1128
Finding a place that accepts my insurance	13.7 (12.2-15.4)	23.4 (21.1-25.9)	14.6 (13.3-15.9)	33.8 (27.8-40.4)	15.6 (14.3-16.9)	45.0 (31.4-59.3)	16.1 (14.8-17.5)	1000
Privacy								
Getting services without telling people you did not want to tell	7.0 (5.9-8.4)	13.3 (11.3-15.5)	8.4 (7.3-9.5)	20.9 (15.9-27.0)	8.9 (7.9-10.1)	34.3 (22.2-48.7)	9.4 (8.3-10.5)	479
Interpersonal relationship barrier								
Going to the clinic because my partner or family member did not want me to go	3.2 (2.5-4.3)	6.8 (5.5-8.5)	3.8 (3.1-4.6)	13.9 (9.8-19.3)	4.1 (3.4-4.9)	31.1 (19.6-45.7)	4.6 (3.8-5.5)	233
Any of the of above barriers	38.7 (36.5-40.9)	55.9 (53.0-58.7)	43.0 (41.2-44.9)	69.0 (62.9-74.5)	44.5 (42.8-46.3)	76.6 (64.6-85.5)	45.1 (43.3-46.9)	2794

^a^ All differences by disability indicator and types of barriers had *P* < .001 according to weighted χ^2^ analyses.

^b^ Some difficulty functioning: some or more difficulty functioning in at least 1 domain.

^c^ Overall disability status: a lot or more difficulty functioning in at least 1 domain.

^d^ Multiple disabilities: a lot or more difficulty functioning in at least 2 domains.

**Table 4.** Associations Between Disability Indicators and Types of Barriers to Reproductive Health (RH) Services in the Past 3 Years Among Survey Participants Who Ever Tried to Access RH (N = 6027)

Types of barriers to RH services in past 3 y	Disability indicator, RR (95% CI)^a^					
	Some difficulty functioning^b^		Overall disability status^c^		Multiple disabilities^d^	
	Unadjusted	Adjusted	Unadjusted	Adjusted	Unadjusted	Adjusted
Logistical	1.46 (1.31-1.62)	1.19 (1.07-1.32)	1.71 (1.48-1.97)	1.32 (1.14-1.52)	2.16 (1.78-2.62)	1.61 (1.33-1.93)
Access	1.60 (1.43-1.80)	1.07 (0.96-1.19)	1.93 (1.67-2.24)	1.21 (1.05-1.39)	2.34 (1.88-2.93)	1.35 (1.12-1.61)
Cost	1.79 (1.58-2.03)	1.26 (1.11-1.42)	2.00 (1.70-2.34)	1.34 (1.15-1.58)	2.24 (1.71-2.93)	1.39 (1.09-1.78)
Privacy	1.89 (1.49-2.39)	1.34 (1.06-1.71)	2.50 (2.86-3.36)	1.52 (1.13-2.03)	3.85 (2.55-5.82)	2.29 (1.52-3.45)
Interpersonal relationship	2.11 (1.48-3.02)	1.69 (1.18-2.41)	3.66 (2.46-5.44)	2.76 (1.84-4.13)	7.60 (4.77-12.13)	4.78 (2.87-7.96)
Any of the barriers	1.44 (1.34-1.56)	1.14 (1.06-1.22)	1.60 (1.46-1.76)	1.21 (1.10-1.33)	1.72 (1.49-1.98)	1.23 (1.08-1.41)

Abbreviation: RR, risk ratio.

^a^ Adjusted for age, race and ethnicity, survey language, US nativity status, educational level, marital status, federal poverty level, geographic region of residence, metropolitan statistical area, LGBTQAI (lesbian, gay, bisexual, transgender, queer [or questioning], asexual [or allied], intersex) identity, pregnancy and abortion history, experiences of medical mistreatment, difficulty trusting health care practitioners, and quality of medical care in the past year. All analyses applied sampling weights.

^b^ Some difficulty functioning: some or more difficulty functioning in at least 1 domain.

^c^ Overall disability status: a lot or more difficulty functioning in at least 1 domain.

^d^ Multiple disabilities: a lot or more difficulty functioning in at least 2 domains.

Two-sided *P* < .05 indicated statistical significance. We conducted all analyses in Stata 17 (StataCorp LLC). We used casewise deletion methods since the rate of missing outcome and covariate data was low at less than 1%.

## Results

A total of 44.6% of adults (6841 of 15 345) and 48.9% of adolescents aged 15 to 17 years (175 of 358) initiated the survey. We excluded 57 people with missing data on all disability or RH access barrier items, leaving a final analytical sample of 6956 people AFAB. Participants had a mean (SD) age of 36.0 (8.3) years and identified as being of Asian, Native Hawaiian, or Pacific Islander (5.7%; 95% CI, 4.9%-6.7%); Black (13.6%; 95% CI, 12.4%-15.0%); Hispanic or Latinx (completed survey in English: 14.9% [95% CI, 13.6%-16.2%]; completed survey in Spanish: 6.5% [95% CI, 5.7%-7.3%]); White (54.7%; 95% CI, 52.9%-56.4%); or other (4.6%; 95% CI, 3.9%-5.5%) race and ethnicity. A total of 46.4% (95% CI, 44.6%-48.1%) of participants had never been pregnant, and 8.5% (95% CI, 7.6%-9.5%) met the WG-SS threshold for disability, of whom 73.2% (95% CI, 67.1%-78.4%) had ever tried to access RH services, with no significant differences by overall disability status (Table 1).

Compared with those without disabilities, participants with disabilities were disproportionately Black individuals (18.8% [95% CI, 14.4%-24.1%] vs 13.2% [95% CI, 11.9%-14.5%]), Latinx individuals (completed survey in English: 18.1% [95% CI, 14.0%-23.0%] vs 14.6% [95% CI, 13.3%-16.0%]; completed survey in Spanish: 8.9% [95% CI, 6.2%-12.8%] vs 6.2% [95% CI, 5.4%-7.1%]), with non–US nativity (19.1% [95% CI, 15.0%-24.0%] vs 14.3% [95% CI, 13.1%-15.6%]), living below the federal poverty level (27.3% [95% CI, 22.3%-32.8%] vs 10.7% [95% CI, 9.7%-11.9%]), members of the LGBTQAI community (16.4% [95% CI, 12.3%-21.6%] vs 11.8% [95% CI, 10.6%-13.1%]), to have ever considered self-managed abortion (4.5% [95% CI, 2.7%-7.4%] vs 2.6% [95% 2.1%-3.3%]), and to have ever experienced medical mistreatment (49.6% [95% CI, 43.7%-55.5%] vs 36.5% [95% CI, 34.8%-38.2%]) (Table 1). Only 2 (communication and multiple disabilities) of the 8 disability indicators differed significantly in ever trying to access RH services (Table 2). Among those who had ever tried to access RH services (n = 6027), across all 8 disability indicators, participants with disabilities experienced more barriers when trying to access RH services than those without disabilities (Figure). People with disabilities vs without disabilities were more likely to experience barriers (69.0% [95% CI, 62.9%-74.5%] vs 43.0% [95% CI, 41.2%-44.9%]), most often logistical (50.7%; 95% CI, 44.2%-57.2%) and access (49.9%; 95% CI, 43.4%-56.4%) barriers. The highest proportions of participants who experienced 3 or more barriers in the past 3 years included those with ADL (75.3%; 95% CI, 61.1%-85.6%), communication (65.1%; 95% CI, 49.5%-78.1%), and multiple (59.9%; 95% CI, 45.6%-72.7%) disabilities and those who met the overall disability status threshold (42.5%; 95% CI, 36.1%-49.1%).

Participants with overall disability status (eg, logistical barriers: 50.7% [95% CI, 44.2%-57.2%] vs 29.7% [95% CI, 28.0%-31.4%]), some difficulty functioning (eg, logistical barriers: 39.1% [95% CI, 36.2%-41.9%] vs 26.7% [95% CI, 24.7%-28.8%]), and multiple disabilities (eg, logistical barriers: 66.2% [95% CI, 53.1%-77.2%] vs 30.7% [95% CI, 29.0%-32.4%]) all experienced significantly more of each type of barrier in trying to access RH services in the past 3 years compared with their counterparts (Table 3). In adjusted and unadjusted regression analyses, participants with overall disability status (eg, logistical barriers: risk ratio [RR], 1.32; 95% CI, 1.14-1.52) and those with multiple disabilities (eg, logistical barriers: RR, 1.61; 95% CI, 1.33-1.93) were significantly more likely to have experienced all types of barriers in trying to access RH services (Table 4). Participants with some difficulty functioning (eg, logistical barriers: RR, 1.19; 95% CI, 1.07-1.32) were significantly more likely to experience each barrier, except for access barriers, which were not statistically significant in adjusted analyses.

## Discussion

In this large national representative survey conducted around the time of the COVID-19 pandemic, 8.5% of participants AFAB of reproductive age met the disability status threshold, of whom over two-thirds experienced barriers to RH access in the past 3 years.^30^ While attempts to access RH services were largely similar by disability status, people with disabilities, particularly those with multiple disabilities, experienced more barriers in trying to access care. Participants with ADL or communication disabilities experienced the greatest number of barriers, with as many as three-quarters experiencing 3 or more barriers in the past 3 years, suggesting that these groups may require the most support. The preponderance of access barriers experienced was consistent with that in other studies that found disparities in general health care access for people with disabilities.^32,33,34^ Thus, disparities in gynecological cancer screenings and contraceptive use may be due to structural barriers to care. Future research needs to examine access to other RH services, such as screening for sexually transmitted infections (STIs) and abortion care, which were not examined in this study, although we did find that people with disabilities are more likely to consider SMA than people without disabilities.

Previous work found that these same barriers to RH access increased from before (2017) to during the COVID-19 pandemic, particularly among people living in poverty and with less formal education.^27^ Thus, the structural barriers to RH care observed in this study may have been compounded during the pandemic, disproportionately affecting people with disabilities. Many factors may explain the barriers experienced during this time, including health risks from COVID-19, lost wages, in-person care restrictions, reduced number of Title X family planning clinics, and other RH-related policy changes.^35^ By assessing the attempts to access RH care, we captured the experiences of people who needed but may have been unable to receive care.

Similar to other work,^36,37^ we found variation in demographic characteristics by disability status. Participants with disabilities were significantly more likely to be Black or Latinx individuals, live below the federal poverty level, and identify as LGBTQAI, and they were much more likely to report experiencing medical mistreatment (eg, being ridiculed, humiliated, or ignored by health care practitioners), receiving fair or poor-quality medical care from their regular physician, and to consider self-managed abortion. These participants may be further marginalized due to ableism, discrimination, limited resources, and intersectional racism and homophobia and thus may encounter additional barriers to care and may experience poor quality and potentially discriminatory health care.

We used multiple categorizations of disability to examine variability in the magnitude and types of barriers experienced by people with a range of disability statuses. We found significant inequities among participants across disability indicators, including those above and below the disability threshold, and all of these participants experienced more of any barrier to RH care compared with those without disabilities. This finding is especially important, as people with some difficulty functioning are often not categorized as having a disability in RH studies.^38^ Efforts to increase the accessibility of RH care must move beyond complying with the ADA standards toward accommodating varying experiences of people with different conditions and levels of functioning.

Participants with disabilities most commonly experienced logistical (ie, finding transportation, getting time off work or school, and finding childcare) and access barriers (eg, finding a place that offers RH services; finding a place where I felt comfortable). A transportation infrastructure that does not meet the needs of people with disabilities likely contributes to inequitable access to care.^39^ Telehealth models of care may help to streamline access and better serve the needs of some people.^40,41^ The high proportion of people who reported difficulty finding a place where they felt comfortable may be associated with previous medical mistreatment and poor-quality health care, and may explain the higher proportion of people with disabilities vs those without disabilities who considered self-managed abortion. Additionally, participants with disabilities also reported difficulty with going to the clinic because their partner or family member did not want them to go. This finding may be explained in part by the greater interdependence between people with disabilities and others for transportation and support with accessing care as well as by the likelihood of people with disabilities to experience intimate partner violence, reproductive coercion, and abuse.^20,26,42,43^ Special attention is needed to address privacy concerns and to identify potential coercion and abuse that might prevent people with disabilities from accessing care.

### Limitations

Findings should be interpreted according to study limitations. First, the measures of access and barriers to care were not comprehensive of all RH services, such as STI screening and abortion care, and did not include all access-related or disability-specific barriers. For example, we did not include barriers related to accessible equipment (eg, adjustable beds), methods of providing information (eg, compatibility of written materials with screen readers), or availability of sign language interpreters. Future research should examine these disability-specific barriers and the implications of abortion bans. Second, we used items from the WG-SS to measure disability, which is known to undercount individuals with chronic or psychiatric conditions,^44^ and we included only 5 of the 6 WG-SS items, limiting the generalizability of the composite measures of disability status and multiple disabilities to other studies. Thus, the disability status prevalence rate of 8.5% was expectedly lower and not directly comparable with other studies using the WG-SS items, which reported an 11% prevalence rate among adult male and female populations, although the domain-specific disability indicators in this study are comparable with those in other studies.^31,45^ Third, given the data collection methods, the study population excluded institutionalized people, which likely left out people who were institutionalized due to their disability. Thus, the estimates of rates of disability and barriers to RH services are not generalizable to institutionalized individuals. Finally, given that we lacked sociodemographic data on those who did not respond to the survey, we were unable to determine the extent to which the sample was biased due to nonresponse. However, the representativeness of the sample was assessed in a previous work, which compared the sociodemographic profile of the current weighted sample to that of the National Survey of Family Growth and found that distributions by age and race and ethnicity were largely similar.^27^ Furthermore, the use of sampling weights reduced some of the bias introduced from unequal selection and nonresponse.

## Conclusions

The findings of this cross-sectional study pointed to large disparities in access to RH care among people AFAB living with disabilities, most of whom experienced multiple barriers to RH care across disability types. While this study did not specifically examine barriers to abortion care, given that people with disabilities were more likely to consider self-managed abortion, restrictions on RH services, including abortion care, are likely to disproportionately affect people with disabilities, further widening health care disparities. The findings highlighted the need to alleviate barriers to RH care, including improving the transportation infrastructure, ensuring the availability of foreign language and sign language interpreters, training practitioners to better serve their patients with disabilities, and reinforcing patient-centered approaches that engender inclusivity in health care settings. There is a continued need for more research to elucidate the unmet RH needs and experiences among people with disabilities.

## Notes

Supplement.
Data Sharing Statement

## References

[1] ADA National Network. What is the definition of disability under the ADA? Accessed June 26, 2023. https://adata.org/faq/what-definition-disability-under-ada

[2] Signore C, Davis M, Tingen CM, Cernich AN. The intersection of disability and pregnancy: risks for maternal morbidity and mortality. J Womens Health (Larchmt). 2021;30(2):147-153. doi:10.1089/jwh.2020.8864 33216671 PMC8020507

[3] Barr JK, Giannotti TE, Van Hoof TJ, Mongoven J, Curry M. Understanding barriers to participation in mammography by women with disabilities. Am J Health Promot. 2008;22(6):381-385. doi:10.4278/ajhp.22.6.381 18677877

[4] Liu SY, Clark MA. Breast and cervical cancer screening practices among disabled women aged 40-75: does quality of the experience matter? J Womens Health (Larchmt). 2008;17(8):1321-1329. doi:10.1089/jwh.2007.0591 18788985 PMC2944439

[5] Armour BS, Thierry JM, Wolf LA. State-level differences in breast and cervical cancer screening by disability status: United States, 2008. Womens Health Issues. 2009;19(6):406-414. doi:10.1016/j.whi.2009.08.006 19879454

[6] Pharr JR, Bungum T. Health disparities experienced by people with disabilities in the United States: a Behavioral Risk Factor Surveillance System study. Glob J Health Sci. 2012;4(6):99-108. doi:10.5539/gjhs.v4n6p99 23121746 PMC4776960

[7] McRee AL, Haydon AA, Halpern CT. Reproductive health of young adults with physical disabilities in the U.S. Prev Med. 2010;51(6):502-504. doi:10.1016/j.ypmed.2010.09.006 20851142 PMC2997130

[8] Wei W, Findley PA, Sambamoorthi U. Disability and receipt of clinical preventive services among women. Womens Health Issues. 2006;16(6):286-296. doi:10.1016/j.whi.2006.09.002 17188212 PMC1937503

[9] Ransohoff JI, Sujin Kumar P, Flynn D, Rubenstein E. Reproductive and pregnancy health care for women with intellectual and developmental disabilities: a scoping review. J Appl Res Intellect Disabil. 2022;35(3):655-674. doi:10.1111/jar.12977 35064736 PMC10119781

[10] Iezzoni LI, Yu J, Wint AJ, Smeltzer SC, Ecker JL. Prevalence of current pregnancy among US women with and without chronic physical disabilities. Med Care. 2013;51(6):555-562. doi:10.1097/MLR.0b013e318290218d 23604018 PMC3733491

[11] Iezzoni LI, Rao SR, Agaronnik ND, El-Jawahri A. Associations between disability and breast or cervical cancers, accounting for screening disparities. Med Care. 2021;59(2):139-147. doi:10.1097/MLR.0000000000001449 33201087 PMC7855335

[12] Ordway A, Garbaccio C, Richardson M, Matrone K, Johnson KL. Health care access and the Americans with Disabilities Act: a mixed methods study. Disabil Health J. 2021;14(1):100967. doi:10.1016/j.dhjo.2020.100967 32768336

[13] Janz HL. Ableism: the undiagnosed malady afflicting medicine. CMAJ. 2019;191(17):E478-E479. doi:10.1503/cmaj.180903 31036612 PMC6488478

[14] Becker H, Stuifbergen A, Tinkle M. Reproductive health care experiences of women with physical disabilities: a qualitative study. Arch Phys Med Rehabil. 1997;78(12 suppl 5):S26-S33. doi:10.1016/S0003-9993(97)90218-5 9422004

[15] Mitra M, Long-Bellil LM, Iezzoni LI, Smeltzer SC, Smith LD. Pregnancy among women with physical disabilities: unmet needs and recommendations on navigating pregnancy. Disabil Health J. 2016;9(3):457-463. doi:10.1016/j.dhjo.2015.12.007 26847669 PMC4903955

[16] Lipson JG, Rogers JG. Pregnancy, birth, and disability: women’s health care experiences. Health Care Women Int. 2000;21(1):11-26. doi:10.1080/073993300245375 11022446

[17] Taouk LH, Fialkow MF, Schulkin JA. Provision of reproductive healthcare to women with disabilities: a survey of obstetrician-gynecologists’ training, practices, and perceived barriers. Health Equity. 2018;2(1):207-215. doi:10.1089/heq.2018.0014 30283869 PMC6110183

[18] Nosek MA, Young ME, Rintala DH, Howland CA, Foley CC, Bennett JL. Barriers to reproductive health maintenance among women with physical disabilities. J Womens Health. 1995;4(5):505-518. doi:10.1089/jwh.1995.4.505

[19] Lehman CA. APN knowledge, self-efficacy, and practices in providing women’s healthcare services to women with disabilities. Rehabil Nurs. 2009;34(5):186-194. doi:10.1002/j.2048-7940.2009.tb00278.x 19772116

[20] Alhusen JL, Bloom T, Laughon K, Behan L, Hughes RB. Perceptions of barriers to effective family planning services among women with disabilities. Disabil Health J. 2021;14(3):101055. doi:10.1016/j.dhjo.2020.101055 33384277 PMC8219807

[21] Horner-Johnson W, Klein KA, Campbell J, Guise JM. Experiences of women with disabilities in accessing and receiving contraceptive care. J Obstet Gynecol Neonatal Nurs. 2021;50(6):732-741. doi:10.1016/j.jogn.2021.07.005 34389287 PMC8759451

[22] Horner-Johnson W, Klein KA, Campbell J, Guise JM. “It would have been nice to have a choice”: barriers to contraceptive decision-making among women with disabilities. Womens Health Issues. 2022;32(3):261-267. doi:10.1016/j.whi.2022.01.001 35148954 PMC9167240

[23] Diab ME, Johnston MV. Relationships between level of disability and receipt of preventive health services. Arch Phys Med Rehabil. 2004;85(5):749-757. doi:10.1016/j.apmr.2003.06.028 15129399

[24] Horner-Johnson W, Kulkarni-Rajasekhara S, Darney BG, Dissanayake M, Caughey AB. Live birth, miscarriage, and abortion among U.S. women with and without disabilities. Disabil Health J. 2017;10(3):382-386. doi:10.1016/j.dhjo.2017.02.006 28431989 PMC5544009

[25] Horner-Johnson W, Dissanayake M, Wu JP, Caughey AB, Darney BG. Pregnancy intendedness by maternal disability status and type in the United States. Perspect Sex Reprod Health. 2020;52(1):31-38. doi:10.1363/psrh.12130 32096336 PMC12331166

[26] García-Cuéllar MM, Pastor-Moreno G, Ruiz-Pérez I, Henares-Montiel J. The prevalence of intimate partner violence against women with disabilities: a systematic review of the literature. Disabil Rehabil. 2023;45(1):1-8. doi:10.1080/09638288.2022.2025927 35038281

[27] Adler A, Biggs MA, Kaller S, Schroeder R, Ralph L. Changes in the frequency and type of barriers to reproductive health care between 2017 and 2021. JAMA Netw Open. 2023;6(4):e237461. doi:10.1001/jamanetworkopen.2023.7461 37036704 PMC10087056

[28] Ipsos. KnowledgePanel: a methodological overview. Accessed June 21, 2023. https://www.ipsos.com/sites/default/files/ipsosknowledgepanelmethodology.pdf

[29] Biggs MA, Ralph L, Raifman S, Foster DG, Grossman D. Support for and interest in alternative models of medication abortion provision among a national probability sample of U.S. women. Contraception. 2019;99(2):118-124. doi:10.1016/j.contraception.2018.10.007 30448203

[30] Washington Group on Disability Statistics. Short set of disability questions. Accessed October 31, 2023. https://www.washingtongroup-disability.com/question-sets/wg-short-set-on-functioning-wg-ss/

[31] Washington Group on Disability Statistics. Creating disability severity indicators using the WG Short Set on functioning (WG-SS). January 5, 2021. Accessed October 31, 2023. https://www.washingtongroup-disability.com/fileadmin/uploads/wg/WG_Document__5H_-_Analytic_Guidelines_for_the_WG-SS__Severity_Indicators_-_CSPro_.pdf

[32] Kennedy J, Wood EG, Frieden L. Disparities in insurance coverage, health services use, and access following implementation of the Affordable Care Act: a comparison of disabled and nondisabled working-age adults. Inquiry. 2017;54:46958017734031. 29166812 10.1177/0046958017734031 PMC5798675

[33] Kaye HS. Disability-related disparities in access to health care before (2008-2010) and after (2015-2017) the Affordable Care Act. Am J Public Health. 2019;109(7):1015-1021. doi:10.2105/AJPH.2019.305056 31095413 PMC6603452

[34] Huang EY, Joo H, Schoo D, Agrawal Y, Chen JX. The impact of hearing loss on health care access during the COVID-19 pandemic. Otolaryngol Head Neck Surg. Published online May 5, 2023. doi:10.1002/ohn.362 37146226

[35] Frederiksen B, Gomez I, Salganicoff A. Rebuilding the Title X network under the Biden Administration. Accessed September 25, 2023. https://www.kff.org/womens-health-policy/issue-brief/rebuilding-the-title-x-network-under-the-biden-administration/

[36] Goyat R, Vyas A, Sambamoorthi U. Racial/ethnic disparities in disability prevalence. J Racial Ethn Health Disparities. 2016;3(4):635-645. doi:10.1007/s40615-015-0182-z 27294757 PMC4919210

[37] Okoro CA, Hollis ND, Cyrus AC, Griffin-Blake S. Prevalence of disabilities and health care access by disability status and type among adults: United States, 2016. MMWR Morb Mortal Wkly Rep. 2018;67(32):882-887. doi:10.15585/mmwr.mm6732a3 30114005 PMC6095650

[38] Wu JP, McKee KS, McKee MM, Meade MA, Plegue MA, Sen A. Use of reversible contraceptive methods among U.S. women with physical or sensory disabilities. Perspect Sex Reprod Health. 2017;49(3):141-147. doi:10.1363/psrh.12031 28514522

[39] Twardzik E, Schrack JA, Pollack Porter KM, Coleman T, Washington K, Swenor BK. TRansit ACessibility Tool (TRACT): developing a novel scoring system for public transportation system accessibility. medRxiv. Preprint posted online March 10, 2023. doi:10.1101/2023.03.07.23286932 PMC10883474 38405233

[40] Pearlman Shapiro M, Myo M, Chen T, Nathan A, Raidoo S. Remote provision of medication abortion and contraception through telemedicine. Obstet Gynecol. 2023;141(6):1056-1061. doi:10.1097/AOG.0000000000005205 37054393

[41] Thompson TA, Sonalkar S, Butler JL, Grossman D. Telemedicine for family planning: a scoping review. Obstet Gynecol Clin North Am. 2020;47(2):287-316. doi:10.1016/j.ogc.2020.02.004 32451019 PMC10093687

[42] Chirwa E, Jewkes R, Van Der Heijden I, Dunkle K. Intimate partner violence among women with and without disabilities: a pooled analysis of baseline data from seven violence-prevention programmes. BMJ Glob Health. 2020;5(11):e002156. doi:10.1136/bmjgh-2019-002156 33208311 PMC7677328

[43] Rodriguez Martinez P. Intimate partner violence experienced by women living with-and without-disability in the European Union. A quantitative intersectional analysis. Front Sociol. 2022;7:948811. doi:10.3389/fsoc.2022.948811 36072499 PMC9443844

[44] Hall JP, Kurth NK, Ipsen C, Myers A, Goddard K. Comparing measures of functional difficulty with self-identified disability: implications for health policy. Health Aff (Millwood). 2022;41(10):1433-1441. doi:10.1377/hlthaff.2022.00395 36190890 PMC10353341

[45] Amilon A, Hansen KM, Kjær AA, Steffensen T. Estimating disability prevalence and disability-related inequalities: does the choice of measure matter? Soc Sci Med. 2021;272:113740. doi:10.1016/j.socscimed.2021.113740 33571943

