# Supplementary material for: Access to Reproductive Health Services Among People With Disabilities
Source: JAMA Netw Open. 2023 Nov 29;6(11):e2344877. doi: 10.1001/jamanetworkopen.2023.44877 (PMC10687653; doi:10.1001/jamanetworkopen.2023.44877)
Supplement: Supplement. — Data Sharing Statement [file jamanetwopen-e2344877-s001.pdf]

Data Sharing Statement

Biggs. Access to Reproductive Health Services Among People With Disabilities. *JAMA Netw Open*. Published November 29, 2023. doi:10.1001/jamanetworkopen.2023.44877

Data
Data available: No

Additional Information
Explanation for why data not available: Given ethical approvals obtained, data will not be posted in a public repository. However, data can be made available upon reasonable request to the corresponding author.
